# The implementation and side effect management of immune checkpoint inhibitors in gynecologic oncology: a JAGO/NOGGO survey

**DOI:** 10.1186/s12885-025-13432-5

**Published:** 2025-01-29

**Authors:** Maximilian Riedel, Helene Herrmann, Thomas Bartl, Anna-Maria Rossner, Anna Tatzber, Chiara Flethe, Dario Zocholl, Barbara Schmalfeldt, Jalid Sehouli, Klaus Pietzner

**Affiliations:** 1 Young Academy of Gynecologic Oncology (JAGO), Nord-Ostdeutsche Gesellschaft für Gynäkologische Onkologie (NOGGO), Berlin, Germany; 2 Department of Obstetrics and Gynecology, TUM University Hospital, Technical University of Munich, Ismaninger Straße 22, D-81675 Munich, Germany; 3 Department of Gynecology and Obstetrics, Heidelberg University Hospital, Heidelberg, Germany; 4 Department of Obstetrics and Gynecology, Division of General Gynecology and Gynecologic Oncology, Comprehensive Cancer Center, Medical University Vienna, Vienna, Austria; 5 Department of Gynecology and Obstetrics, Gynecologic Oncology, St. Josefs-Hospital Wiesbaden GmbH, Affiliated Hospital of Medical University of Mainz, Mainz, Germany; 6 Breast Center, Campus Mitte, Charité-Universitätsmedizin Berlin, Corporate Member of Freie Universität Berlin, Humboldt-Universität zu Berlin, Berlin Institute of Health, Berlin, Germany; 7 Department of Gynecology, Center for Oncological Surgery, Campus Virchow Klinikum, Charité-Universitätsmedizin Berlin, Corporate Member of Freie Universität Berlin, Humboldt- Universität zu Berlin, Berlin Institute of Health, Berlin, Germany; 8 Institute of Biometry and Clinical Epidemiology, Charité Medical University Berlin, Corporate Member of Freie Universität Berlin and Humboldt-Universität zu Berlin, Berlin, Germany; 9 Department of Gynecology and Gynecologic Oncology, University Medical Center Hamburg- Eppendorf, Hamburg, Germany

**Keywords:** Gynecologic oncology, Breast cancer, Immune checkpoint blockade, Immune adverse events

## Abstract

**Background:**

The integration of immune checkpoint inhibitors (ICIs) into routine gynecologic cancer treatment requires a thorough understanding of how to manage immune-related adverse events (irAEs) to ensure patient safety. However, reports on real-world clinical experience in the management of ICIs in gynecologic oncology are very limited. The aim of this survey was to provide a real-world overview of the experiences and the current state of irAE management of ICIs in Germany, Switzerland, and Austria.

**Methods:**

We designed a questionnaire consisting of 34 items focused on physicans’ clinical experiences with ICIs and their management of irAEs. The survey was distributed between October 2022 and May 2023 to medical professionals with experience in the field of gynecologic oncology.

**Results:**

A total of 221 gynecologists participated in the study. Most respondents (*n* = 130, 59.1%) were primarily engaged in gynecologic oncology at the time of the survey, with an average of ten years of clinical experience. Individual experiences with regard to irAEs varied significantly. When asked which irAEs they had observed “frequently” or “very frequently”, respondents most commonly reported thyroiditis (37.2%), followed by skin reactions (23.6%), and pneumonitis (10.6%). A total of *n* = 16 (7.4%) reported at least one death of a patient due to irAEs. Feeling “unconfident” or “very unconfident” about managing irAEs was reported by 35.6% (*n* = 78). With regard to clinical management of adverse events after discontinuation of treatment, 32.4% (*n* = 68) ceased to inquire about irAEs after six months.

**Conclusion:**

The results of this survey provide valuable insights into physicians’ real-world experiences with irAEs associated with ICI treatment. Dealing with serious immune-related and potentially life-threatening side effects has become a routine aspect of clinical practice. Many physicians, however, express a lack of sufficient familiarity with irAEs and their management. Therefore, it is essential to improve medical education, specialized oncological training, and close interdisciplinary collaboration to improve patient care.

**Supplementary Information:**

The online version contains supplementary material available at 10.1186/s12885-025-13432-5.

## Introduction

Immune checkpoint inhibitors (ICIs) have led to significant clinical benefits in gynecologic oncology. Ever since their first introduction for treating metastatic or locally advanced triple-negative breast cancer, ICIs have significantly expanded their footprint within the field of gynecologic oncology [1]. EMA-approvals for the first-line treatment of advanced or metastatic endometrial cancer [2], PD-L1 positive metastatic cervical cancer [3], or the (neo-) adjuvant treatment of high-risk triple-negative breast cancer followed [4]. With numerous studies recently completed or ongoing across all sorts and stages of gynecologic malignancies, the number of patients receiving ICI therapy is expected to rise significantly in the near future.

The most common mechanism of action of ICIs is based on the release of the “immunologic break” between cytotoxic T cells and cancer cells by breaking up the PD-1/PD-L1 receptor-ligand interaction [5]. However, this can also trigger non-specific immune reactions associated with a novel array of immune-related adverse events (irAEs) that have been previously unfamiliar to gynecologic oncologists. These may include, but are not confined to, skin reactions like maculopapular exanthema [6], endocrinopathies like thyroiditis or hypothyroidism [7], or auto-immune reactions against various organs, including pneumonitis, hepatitis, or colitis [8]. These effects may occur several months after the cessation of treatment, often with subtle and non-specific clinical manifestations requiring special clinical awareness [9]. Therefore, significant diagnostic and therapeutic adaptations of clinical management are necessary. This may involve longer post-therapeutic clinical evaluations to monitor for irAEs and re-evaluate screening tools, such as routine lab tests that include thyroid hormones, liver function, or cardiac output parameters [10]. Another relevant aspect in the context of immune checkpoint blockade is the so-called pseudoprogression which incidence varies in literature between 0 and 15% [11]. This term refers to a phenomenon where tumors initially appear to grow or new lesions appear on imaging scans before a subsequent reduction in tumor size occurs indicating tumor response. This apparent worsening can be misleading and is caused by immune cell infiltration and inflammation within the tumor rather than true tumor growth.

Globally, oncology subspecialties are distinctly classified, and breast cancer is typically managed as a separate discipline from gynecologic oncology which primarily focuses on ovarian, uterine, and cervical cancers. However, in Germany, Austria, and Switzerland, breast cancer is usually considered an integral part of gynecologic oncology, with many gynecologic oncologists actively involved in the diagnosis and treatment of breast cancer across both the primary and the metastatic setting. This integrated approach may not only expose gynecologic oncologists in those countries to a wider range and higher frequency of irAEs, but it also underscores the importance of comprehensive education and the establishment of clear management protocols to ensure optimal patient care.

International real-world-data from dedicated surveys dealing with the practical experiences and the management of irAEs are very limited. This lack of data is particularly evident in the field of gynecologic oncology, where real-world information on the management of immune checkpoint blockade and the subsequent autoimmune side effects are currently absent. Given the transformative impact of ICIs in gynecologic oncology, it is crucial to understand how well the medical community, specifically the German-speaking gynecologists in our study, is adapting to these changes. To the best of our knowledge, this is the first survey directly aimed at investigating their comfort and proficiency in administering these treatments, their experiences and challenges in managing associated irAEs, and specific areas where they feel that additional support or training is needed. This survey was intended to provide an overview of the current state of real-word practice and highlight areas for potential improvement.

## Methods

### Data distribution and acquisition

This survey was designed and conducted by the *Young Academy of Gynecologic Oncology* (JAGO) under supervision of the *North-Eastern German Society of Gynecologic Oncology* (NOGGO). The study protocol was approved by the ethics committee of Charité-Universitätsmedizin Berlin and registered under the license EA1/198/22. An online multiple-choice questionnaire was established to explore clinical experiences and management strategies for ICI therapy. The survey questionnaire included 34 items, of which 30 were dichotomous or classification questions, while 4 utilized either a numeric rating scale or a five-point Likert scale to demonstrate participants’ agreement or disagreement with the specific statements. The questionnaire was divided into seven sections: (I) demographic data (six questions), (II) general experience with ICI therapy (four questions), (III) experience with irAEs following ICI therapy (four questions), (IV) management of irAEs following ICI therapy (five questions), (V) level of knowledge and training in managing irAEs (eight questions), (VI) long-term follow-up and management of side effects after the cessation of ICI therapy (four questions), and (VII) the role of pseudoprogression in the therapy of ICIs (three questions). For the full survey and list of questions, see the supplementary material.

The questionnaire was distributed through various channels from October 2022 to May 2023. These included a face-to-face dissemination at the congress of the *German Society of Gynecology and Obstetrics* (DGGG) held from October 13–15, 2022, in Munich (Germany) with about 5000 attendees, and publication in the German gynecology-related journal “Frauenarzt”. This journal is the official publication organ of the *Professional Association of Gynecologists* (BVF) and the leading clinical journal for gynecologists in Germany with a circulation of approximately 23.800. The article by Bartl et al. dealt with the application of ICIs in gynecologic oncology and included a QR code providing access to our survey [10]. Moreover, various online invitations via email along with a QR code to academic and non-academic OB/GYN institutions in Germany, Switzerland and Austria, to the NOGGO mailing list and the JAGO alumni mailing list were issued. The survey targeted gynecologists from Germany, Austria, and Switzerland across all clinical positions who were either currently treating or had previously treated patients with gynecologic malignancies (including breast cancer), regardless of their level of experience with ICIs. Because our survey employed an open invitation approach, including dissemination through a widely circulated German OB/GYN journal, we are unable to determine a specific response rate.

Survey data collection was facilitated using SurveyMonkey (Survey Monkey Inc., San Mateo, CA, USA). Participation was entirely voluntary; all data were anonymized. Participants provided electronic informed consent to participate and agreed electronically to data analysis. No compensation was offered for participation.

### Statistical analysis

Tables were generated using Word (version included in Office 2405, 2024, Microsoft). Figures and statistical analysis were generated in Prism (version 10.3.1, 2024, GraphPad). Descriptive statistics were calculated for each individual item, including means and relative values. Chi-square test was used to evaluate the level of confidence (confident/very confident vs. unconfident/very unconfident) between the experienced (clinical directors and consultants) and less experienced (residents and specialized OB/GYN physicians) subgroups of respondents. Unpaired t-test was used to analyze the numbers of irAEs reported by the different sub-groups (clinical directors, consultants, specialized OB/GYN physicians, and residents) of respondents. All *p*-values < 0.05 were defined as statistically significant.

## Results

### Demographics and general experiences with ICIs

**Fig. 1:**
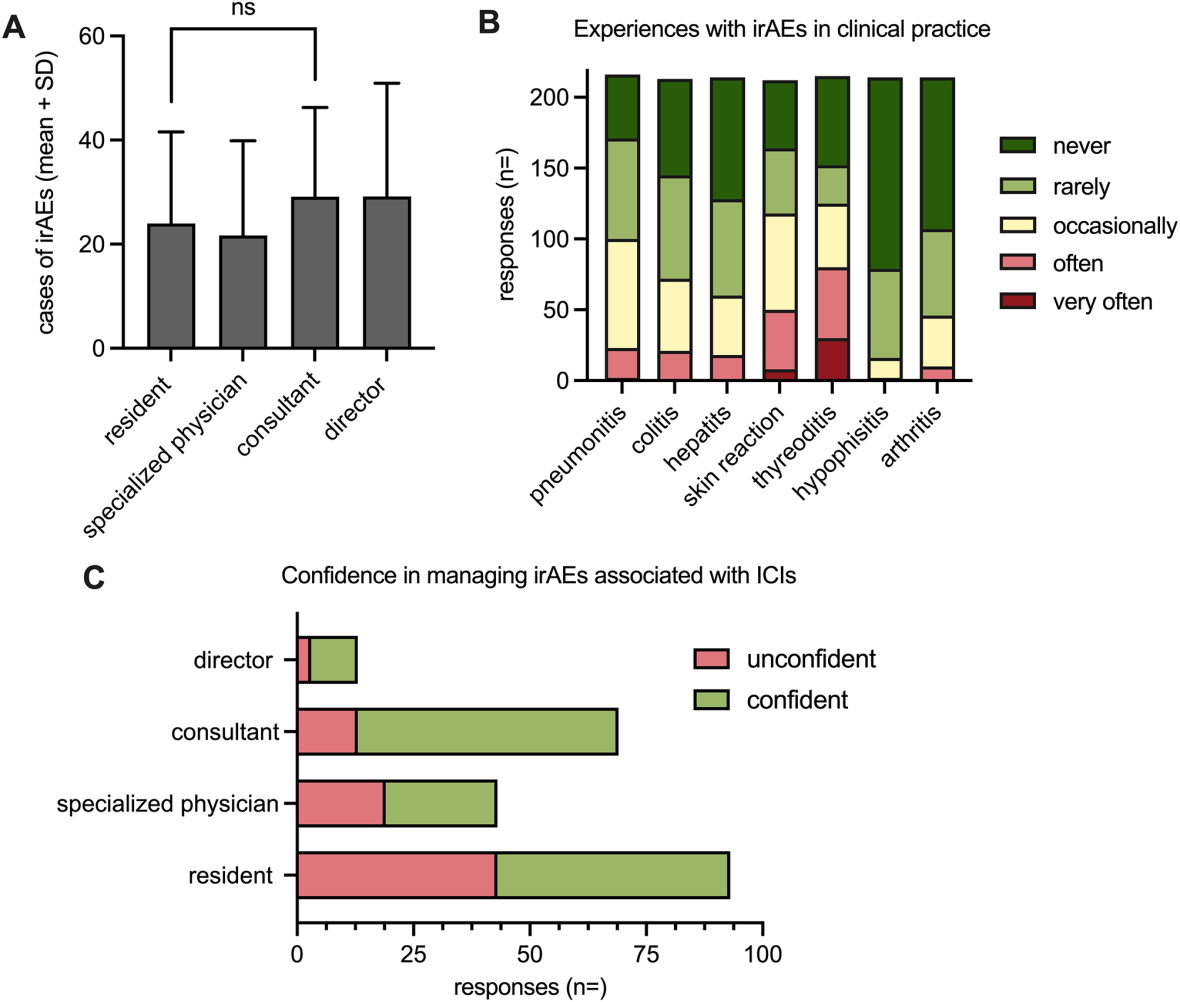
(**A**) Mean cases (+ standard deviation) of irAEs experienced by residents, specialized physicans in OB/GYN, consultants, and clinical directors. Unpaired t-test was used for statistical testing (ns = not significant). (**B**) Number of responses in different colors from “never” to “very often” with regard to the frequency of cases of irAEs associated with ICIs experienced in clinical practice. (**C**) Responses from residents, specialized physicians in OB/GYN, consultants and clinical directors with regard to their level of confidence (light green: “confident” or “very confident”; light red: “unconfident” or “very unconfident”) managing irAEs associated with ICIs

A total of *n* = 221 respondents actively participated in our survey. A comprehensive list of the states of origin of the respondents can be found in Supp. Table 1. Among these, a majority (59.1%) was at the time of the survey actively occupied in the field of gynecologic oncology and reported a mean professional expertise of ten years. Most held senior positions as consultants or clinical directors in obstetrics and gynecology (OB/GYN), were primarily affiliated with university hospitals, and were predominantly from Germany (Table 1). On average, the respondents have treated a median number of *n* = 25 patients (95% CI, *n* = 4–50) with ICIs over the course of their clinical careers. No significant differences were observed when comparing these numbers for the sub-groups of residents, specialized physicians, consultants, or directors (Fig. 1A). A majority (96.4%; *n* = 212) anticipated an increase in the number of patients receiving ICI therapy in the future, while only 3.6% (*n* = 8) expected the usage to remain stable or decrease.

**Table 1:** Characteristics of study participants

Participants in total	*n* = 221	
**Currently occupied in the field of**		
- gynecologic oncology	59.1% (*n* = 130)	
- general gynecology	34.1% (*n* = 75)	
- other	6.8% (*n* = 15)	
**Clinical position**		
- resident	41.9% (*n* = 92)	
- specialized physician in OB/GYN	19.5% (*n* = 43)	
- consultant	31.8% (*n* = 70)	
- clinical director	5.9% (*n* = 13)	
- none of the above	0.9% (*n* = 2)	
**Average professional experience (+ standard deviation)**	10 years (+/- 9 years)	
**Affiliation**		
- university hospital	54.2% (*n* = 118)	
- accredited tumor center distinct from university hospitals	30.7 (*n* = 67)	
- ambulant practice	10.1% (*n* = 22)	
- non-accredited peripheral clinic	4.1% (*n* = 9)	
- other	0.9% (*n* = 2)	
**Country of origin**		
- Germany	85.5% (*n* = 188)	
- Austria	12.7% (*n* = 28)	
- Switzerland	1.8% (*n* = 4)	
**Tumor entities treated with ICIs (multiple answers)**		
- breast cancer	77.9% (*n* = 169)	
- endometrial cancer	61.3% (*n* = 133)	
- cervical cancer	60.4% (*n* = 131)	
- ovarian cancer	46.5% (*n* = 101)	
- vulvar cancer	21.7% (*n* = 47)	
- choriocarcinoma	4.6% (*n* = 10)	
- others	4.1% (*n* = 9)	
- none of the mentioned above	7.4% (*n* = 16)	

### Experiences with side effects of ICI therapy

A broad spectrum of side effects has been reported following ICI therapy with most occurring at low to moderate frequency levels. When asked which irAEs they had observed “frequently” or “very frequently”, respondents most often cited thyroiditis (37.2%) followed by skin reactions (23.6%). Less common side effects involved conditions such as pneumonitis (10.6%), colitis (9.9%), hepatitis (8.4%), arthritis (4.7%), or hypophysitis (0.9%) (Fig. 1B). Over 80% have – on at least one occasion – admitted or referred a patient for inpatient treatment due to harmful side effects originating from immune checkpoint blockade (Table 2). A significant portion (33.6%, *n* = 73) has encountered clinical situations that necessitated intensive care interventions; a smaller subset (7.4%, *n* = 16) reported even cases of patient mortality due to severe side effects directly linked to ICIs.

**Table 2:** How often have you admitted or referred a patient for inpatient treatment due to irAEs from ICI therapy?

	%	*n*=
**0 times**	20.6%	45
**1–5 times**	53.7%	117
**6–10 times**	15.6%	34
**11–20 times**	6.9%	15
**21–30 times**	2.3%	5
**more than 30 times**	0.9%	2

### Management of side effects of ICI therapy

Regarding the management of irAEs following immune checkpoint blockade, 29.2% (*n* = 64) expressed a moderate to high level of confidence, while 35.6% (*n* = 78) reported feeling “unconfident” or “very unconfident”. When comparing confidence levels in treating patients with ICIs, respondents with greater general clinical experience and higher clinical rank demonstrated markable higher confidence levels compared to less experienced individuals (Fig. 1C). Using Chi-square test, we identified a significant difference (*p* < 0.0001; df = 1; χ^2^ = 14.2) in the level of confidence (confident/very confident vs. unconfident/very unconfident) between the experienced (clinical directors and consultants) and less experienced (residents and specialized OB/GYN physicians) subgroups of respondents. When asked about the latest possible timing of clinical manifestations of irAEs after the cessation of ICI therapy, responses varied from “a few days” (3.8%; *n* = 8), “a few weeks” (13.8%; *n* = 29), “a few months” (44.8%; *n* = 94) up to “a few years” (37.6%; *n* = 79). We asked the respondents to provide a comprehensive list of laboratory parameters that are routinely analyzed in patients undergoing ICI therapy (Table 3). Most respondents (79.5%; *n* = 171) have already utilized cortisone to address irAEs. Moreover, 72.2% (*n* = 151) expressed the opinion that the use of cortisone does not negatively impact the oncological efficacy of ICIs.

**Table 3:** Which laboratory values do you routinely analyze under ICI therapy (multiple answers possible)?

	%	*n*=
**Differential blood count**	80.8	173
**Creatinine**	88.3	189
**Thyroid stimulating hormone (TSH)**	82.2	176
**Aspartate Aminotransferase (AST) / Alanine Aminotransferase (ALT)**	86.4	185
**Blood glucose**	43.0	92
**Electrolytes**	74.8	160
**Glycated hemoglobin (HbA1c)**	28.5	61
**Troponin**	9.8	21
**C-reactive protein (CRP)**	37.8	81
**Bilirubin**	63.1	135
**Amylase / Lipase**	49.5	106
**Cortisol**	24.3	52
**Adrenocorticotropic hormone (ACTH)**	16.8	36
**Creatine kinase (CK) / Creatine kinase-MB (CK-MB)**	15.4	33
**None of the mentioned above**	5.6	12
**Others**	8.4	18

### Long-term follow-up and management of irAEs after discontinuation of ICI therapy

A majority (73.5%; *n* = 155) reported that they systematically inquire about symptoms indicative of irAEs during follow-up consultations. However, 32.4% (*n* = 68) would stop this practice after six months following the discontinuation of therapy (Supp. Table 2). To enhance patient awareness, strategies such as the use of informed consent forms (80.1%, *n* = 169) or brochures from pharmaceutical manufacturers (50.2%, *n* = 106) were most commonly used.

### The role of pseudoprogression in ICI therapy

The term pseudoprogression was recognized by 65.0% (*n* = 141). Nearly half of the respondents (49.0%; *n* = 99) have not encountered any occurrences of pseudoprogression in their clinical practice with ICI therapy (Table 4). When confronted with a suspicion of progress in the “target lesion” shortly after initiating ICI therapy based on imaging results, 94.8% (*n* = 200) would opt to continue ICI therapy and re-assess the situation within a period of six weeks to three months.

**Table 4:** How many instances of pseudoprogression have you encountered in your clinical practice under ICI therapy?

	%	*n*=
**0 times**	49.0	99
**1 times**	17.8	36
**2 times**	14.4	29
**3 times**	6.9	14
**4 times**	3.0	6
**5 times**	2.5	5
**more than 5 times**	6.4	13

### Education and training on side effect management with ICIs

Most institutions have standard operating procedures (SOPs) for managing irAEs either already in use (39.4%; *n* = 84) or currently under development (22.5%; *n* = 48). Perspectives on the accessibility of crucial information for managing irAEs varied widely. While 18.2% (*n* = 39) found access to pertinent information “difficult” or “very difficult”, 37.0% (*n* = 80) considered it “easy” or “very easy”. Approximately half (53.2%; *n* = 116) have been engaged in dedicated training specifically focusing on the management of irAEs. With regard to the preferences for informational resources of side effect management, usage patterns varied significantly. Mobile apps and SOPs were frequently utilized, whereas side effect registries and in-house training were less commonly relied upon (Supp. Table 3).

## Discussion

### Summary of main results

This study offers real-world data on the experiences and challenges faced by gynecologic oncologists when managing irAEs associated with immune checkpoint blockade. Given the diverse range of respondents from various states in Germany, Austria, and Switzerland, a significant level of representability and generalizability, especially for oncological centers, can be inferred. Personal experiences in clinical practice with common irAEs, such as thyroiditis and skin reactions, are consistent with published data. Notably, there were also reports of critical side effects, including patient mortality. We reveal a considerable variability in the confidence levels and clinical practices, with many clinicians expressing uncertainty in handling irAEs.

### Results in the context of published literature

The reasons behind the varying confidence levels and diverse practices among clinicians in managing irAEs are probably multifaceted. One factor might be the level of medical training among the respondents. In our cohort of respondents, we had a subset of likely well-experienced physicians, ranging from OB/GYN specialists to consultants and clinical directors, who made up more than half of the respondents. The remaining 41.9% consisted of trainees (residents) in OB/GYN with probably varying levels of experience. However, with an average work experience of ten years among all respondents, our cohort can generally be regarded as highly experienced. Another factor may be the relatively recent introduction of ICIs in the clinical routine of gynecologic oncologists resulting in a still limited pool of experience and knowledge [5]. International reports or surveys on the practical management of irAEs in gynecologic malignancies are extremely limited. In fact, we found no real-world data from surveys specifically investigating experiences with ICIs in gynecologic oncology. Based on the existing literature, the medical community’s overall experience with irAEs and their management seems somewhat limited. A 2022 Italian survey by Giamello et al. involving 132 emergency physicians revealed that they did not feel sufficiently knowledgeable about ICIs and irAEs. Only a small proportion would recommend consulting an oncologist for further advice when treating a patient receiving ICIs, and 20% did not consider irAEs as part of the differential diagnosis for such patients [12]. In a similar survey by Khalid et al., involving 155 physicians specializing in internal, family, or emergency medicine, significant knowledge gaps were identified in the management of irAEs. The study revealed common misconceptions about ICIs, along with low confidence among physicians in recognizing and managing irAEs [13].

After earning a medical license in Germany, physicians complete a five to six-year residency program in OB/GYN, during which they gain extensive clinical experience in various aspects of women’s health, including surgery, clinical oncology, reproductive medicine, and prenatal care [14]. The additional training for sub-specializing in gynecologic oncology, similar to fellowship programs in the international context, typically takes a minimum of 24 months. From an international point of view, it is crucial to note that breast cancer treatment is fully integrated within the field of OB/GYN in German-speaking countries. This includes diagnosis, surgery, and systemic therapy both in the primary and in the metastatic setting. Thus, gynecologists may have exposure to a significantly higher number of cases involving ICIs, as they treat both breast and gynecologic cancer patients. In comparison, in a lot of other health care systems, patients with breast cancer and gynecologic malignancies are treated by separate specialties, especially the systemic treatment of breast cancer is separated from OB/GYN. Therefore, in medical systems where gynecologic oncologists do not treat breast cancer patients, there may be an even greater lack of exposure to patients receiving immune checkpoint blockade, leading to a less steep learning curve in managing irAEs. Moreover, German gynecologic oncologists often combine roles as both surgeons and providers of systemic therapies, including immunotherapy, which reflects an integrated approach to patient care. This dual responsibility allows for a seamless transition between surgical and systemic treatment modalities, enabling a comprehensive clinical management, especially with regard to post-neoadjuvant therapeutic algorithms in breast cancer.

The potentially delayed onset of irAEs introduces an additional layer of intricacy in patient management. However, there are currently no universally accepted definitions of chronic or delayed irAEs [15]. In a retrospective study by Patrinely et al. focusing on melanoma patients treated with adjuvant anti-PD-1 therapy, chronic irAEs were defined as those persisting for 12 weeks or more after treatment cessation. In line with this definition, Johnson et al. reported that approximately 43% of patients would experience at least one chronic irAE [16]. Patrinely at al. also suggested that alternative definitions, particularly for longer-term effects, should be considered [15]. In another observational study, Owen et al. analyzed data from 20 oncologic centers and reported that 118 patients developed 140 irAEs related to ICI therapy, with an estimated incidence of 5.3%. These irAEs included colitis, rash, and pneumonitis and typically occurred 16 months (ranging from 12 to 53 months) after the start of treatment. Most of these patients were still receiving ICIs at the onset of the irAEs; however, 16 patients (14%) experienced irAEs more than three months after discontinuing ICIs. While the vast majority of irAEs (161 cases; 96.4%) were mild (grade 1 or 2), two deaths were attributed to these side effects, underscoring the severity and complexity of managing late-onset irAEs [17]. When administering ICIs, long-term vigilance and a proactive approach to patient follow-up are necessary to ensure that any late-emerging side effects are promptly identified and adequately addressed. In our study, the clinical knowledge of the respondents about the immune-related spectrum of side effects was definitely present and appreciated. However, it is concerning that approximately a third of our survey’s respondents declared that they ceased to ask about potential symptoms of immune-related side effects already six months after the end of treatment. This practice may potentially lead to the oversight or misinterpretation of potential harmful delayed irAEs.

The types and frequencies of irAEs observed in our study from ICI therapy confirm what is known from the existing literature [18]. The respondents from our survey reported thyroiditis and skin reactions as the most common irAEs, ranking them higher than pneumonitis, colitis, hepatitis, arthritis, or hypophysitis. These findings align with the existing literature. Shehaj et al. conducted a retrospective review of patients with various gynecologic malignancies treated with ICIs at their gynecologic cancer center in Germany from 2018 to 2023. They reported thyroiditis as the most prevalent irAE, followed by hepatitis, colitis, and pneumonitis [19]. Similarly, Yin et al. identified skin- and endocrine-related irAEs the most prevalent in their systematic review of clinical trial data [20]. Most irAEs are mild to moderate and can be managed with treatment discontinuation or intermediate immunosuppression. However, the significant number of patients admitted or referred for inpatient care due to irAEs, as reported in our study, along with cases requiring intensive care and even patient death, highlight the critical importance of expert management of these side effects. The fact that 7.4% of our respondents reported cases of patient mortality linked to ICIs may only initially seem high but reflects the serious risks associated with these treatments. In a retrospective analysis, Wang et al. examined over 16 million adverse drug reactions from the WHO’s Vigilyze database and records from seven academic centers focusing on patients treated with ICIs. They identified 613 fatal events reported worldwide between 2009 and 2018. The causes of death varied by treatment type: colitis was frequently associated with anti-CTLA-4 related fatalities, while pneumonitis, hepatitis, and neurotoxic effects were more common in anti-PD-1/PD-L1 related deaths. A meta-analysis of 112 trials, encompassing 19,217 patients undergoing ICI therapy, found that toxicity-related fatality rates ranged from 0.36 to 1.23%, with myocarditis showing the highest mortality among reported cases [21].

### Limitations

One of the key strengths of this study is its comprehensive examination of real-world experiences among gynecologic oncologists in managing ICIs and their associated irAEs. However, the overrepresentation of participants from university hospitals may reduce the generalizability of the findings to less specialized clinics. The questionnaire used was not previously validated which could impact the reliability of the results. Furthermore, the survey largely reflects the experiences of physicians with a solid background in gynecologic oncology, therefore, it surely excludes the perspectives of general practitioners or gynecologists in the outpatient setting with less exposure to cancer patients.

### Implications for practice and future research

The broad spectrum of side effects, ranging from common issues like thyroiditis and skin reactions to rarer but serious conditions such as hypophysitis and myocarditis, highlights the critical need for thorough education and training. This knowledge is crucial for ensuring the timely recognition and effective management of these potentially life-threatening adverse events or the prevention of harmful clinical decisions with regard to phenomena like pseudoprogression. The success of specialized training is exemplified by a study by Offner and Rinke among oncology nurses. In their pilot study, they focused on assessing the confidence levels of nurses in managing the complex treatment paradigm of immuno-oncology. Using the specifically developed Oncology Nurse Immunotherapy Confidence Survey, which included 28 confidence measurements along with demographic data collection and a brief quasi-thematic analysis, the study found a significant 51% overall improvement in nurse confidence levels following specific educational interventions [22].

It is crucial to emphasize that our recommendations are not only intended for providers working in specialized environments as gynecologic oncologists but also for general OB/GYN practitioners. This broader focus is essential for two reasons. First, gynecologic oncologists who directly administer ICIs must possess a robust theoretical understanding and hands-on experience to manage irAEs effectively. Second, irAEs can present subtly and unpredictably, with some treatments extending over years. This means that clinical practitioners in smaller hospitals, outpatient clinics, or private practices must also be adequately trained to recognize (early) signs of irAEs. While these practitioners may not manage irAEs directly, their ability to identify potential complications and ensure timely referral to specialized centers is nonetheless critical for patient safety. Therefore, the educational content must be tailored to equip all levels of OB/GYN practitioners with the knowledge and skills necessary to identify, address, and appropriately escalate care for irAEs, ensuring comprehensive and effective management across all healthcare settings.

Implementing the recommendations from guidelines by reputable organizations like ESMO [23] or ASCO [18] can help standardize the management of irAEs and improve patient outcome. Based on these guidelines, developing a repository of in-house resources and tools that clinicians can readily access for guidance can significantly contribute to boost their confidence and competence in managing irAEs. The limited training on irAEs among participants is a concerning finding, particularly given the potentially life-threatening nature of these events. Our data indicate that only half of the respondents reported having undergone specific training related to irAE management. This highlights a critical gap in clinicial education and preparedness which could directly impact patient safety and outcomes. Potential reasons for this disparity may include variations in institutional training programs, insufficient integration of irAE-related topics into medical education, or limited awareness of the critical importance of irAEs in clinical practice. Within our cohort of respondents, a significant proportion held senior clinical positions. It is plausible that individuals in these roles may demonstrate less willingness to engage in structured training programs, particularly when compared to residents in training who are generally more accustomed to formal education. However, the fact that a majority of our survey respondents stated that their institutions have already implemented or are currently implementing SOPs for the management of irAEs is therefore very positive. Additionally, establishing multidisciplinary teams from various specialties, such as pulmonology, nephrology, and dermatology, or centralizing patients undergoing ICI therapy in dedicated oncologic centers, could streamline the management of side effects and ultimately improve patient outcomes. Broader studies that encompass diverse clinical settings and include a wider range of healthcare providers could help address the limitations of this study and provide a more comprehensive understanding of ICI management across various healthcare environments.

## Conclusion

As our real-world data demonstrate, ICIs are associated with serious immune-related and potentially life-threatening side effects. However, a significant number of physicians expressed a lack of familiarity with these side effects and their management, highlighting the need for attention to this issue. Therefore, it is crucial to provide specialized OB/GYN training and interdisciplinary collaboration to improve the management of these side effects. By doing so, we can help ensure that patients fully benefit from these groundbreaking therapies while minimizing risks to their quality of life and overall well-being.

## Electronic supplementary material

Below is the link to the electronic supplementary material.

Supplementary Material 1

Supplementary Material 2

## Data Availability

Data are available upon reasonable request from: klaus.pietzner@charite.de.
