# Supplementary material for: The implementation and side effect management of immune checkpoint inhibitors in gynecologic oncology: a JAGO/NOGGO survey
Source: BMC Cancer. 2025 Jan 29;25:170. doi: 10.1186/s12885-025-13432-5 (PMC11776233; doi:10.1186/s12885-025-13432-5)
Supplement: Supplementary file 1 — Supplementary Material 1 [file 12885_2025_13432_MOESM1_ESM.docx]

| 1. **In which clinical area are you currently occupied?**   - Gynecology  - Gynecologic oncology  - Other |
| --- |
| 1. **How many years of professional experience do you have?**   - “free text” |
| 1. **What is your clinical position?**   - Resident  - Specialized physician in OB/GYN  - Consultant  - Director  - None of the above |
| 1. **In which country are you currently working?**   - Germany  - Switzerland  - Austria |
| 1. **In which federal state (Germany, Austria) or Canton (Switzerland) do you operate?**   - “free text” |
| 1. **Which institution do you currently work for?**  - University hospital - Accredited tumor center distinct from university hospitals - Ambulant practice - Non-accredited peripheral clinic - Other |
| 1. **How many patients have you treated with ICI so far?**  - “free text” |
| 1. **How do you think the number of patients undergoing ICI therapy will develop at your institution in the future?**  - Significant increase - Small increase - Stable - Small decrease - Significant decrease |
| 1. **Which tumor entities have you already treated with ICIs (multiple answers possible):**  - Breast cancer - Endometrial cancer - Cervical cancer - Ovarian cancer - Vulvar cancer - Choriocarcinoma - Others: “free text“ - None of the mentioned above |
| 1. **In which clinical setting have you used ICIs so far (multiple answers possible)?**  - According to EMA (European Medicines Agency) approval - As “off-label use” - In the context of clinical drug trials - Other: “free text” - None of the above |
| 1. **Which laboratory values do you routinely analyze under ICI therapy (multiple answers possible)?**  - Differential blood count - Creatinine - Thyroid stimulation hormone (TSH) - Aspartate Aminotransferase (AST) / Alanine Aminotransferase (ALT) - Blood glucose - Electrolytes - Glycated hemoglobin (HbA1c) - Troponin - C-reactive protein (CRP) - Bilirubin - Amylase/Lipase - Cortisol - Adrenocorticotropic hormone (ACTH) - Creatine kinase (CK)/ Creatine kinase-MB (CK-MB) - None of the mentioned above - Others |

| 1. **How often have you admitted or referred a patient for inpatient treatment due to irAEs following ICI therapy?**   - 0 times  - 1-5 times  - 6-10 times  - 11-20 times  - 21-30 times  - More than 30 times |
| --- |
| 1. **Has intensive care treatment already been necessary for one of your patients due to serious side effect following ICI therapy?**  - Yes - No |
| 1. **Have you experienced a patient’s death as a direct result of side effects following ICI therapy?**  - Yes - No |
| 1. **How confident do you feel about managing irAEs as a result of ICI therapy?**   - Very confident  - Confident  - Undecided  - Unconfident  - Very unconfident |
| 1. **How often have you experienced the following irAEs of ICIs in your clinical routine?**   - Pneumonitis (very often, often, occasionally, rarely, never)  - Colitis (very often, often, occasionally, rarely, never  - Hepatitis (very often, often, occasionally, rarely, never  - Skin reaction (very often, often, occasionally, rarely, never)  - Thyroiditis (very often, often, occasionally, rarely, never)  - Hypophysitis (very often, often, occasionally, rarely, never)  - Arthritis (very often, often, occasionally, rarely, never) |
| 1. **What would you do if - shortly after starting treatment with checkpoint inhibitors - imaging shows a suspected progression in the size of the target lesion?**   - Immediate change of therapy  - Continue therapy and re-evaluation after 6 weeks  - Continue therapy and re-evaluation after 3 months  - Continue therapy and re-evaluation after 6 months  - Continue therapy and re-evaluation after more than 6 months |
| 1. **Have you ever used cortisone to treat irAEs from ICIs?**   - Yes  - No |
| 1. **In your opinion, can the use of cortisone have a negative impact on the oncological efficacy of ICIs?**   - Yes  - No |
| 1. **In your opinion, how long after the end of treatment with ICIs can treatment-related side effects be expected?**   - A few days  - A few weeks  - Up to several months  - Up to several years |
| 1. **Does your institution have standard operating procedures (SOPs) for dealing with irAEs in the context of ICI therapy?**   - Yes  - No  - Currently in progress |
| 1. **In general, how do you rate the access to relevant information on the management of irAEs of ICIs?**   - Very difficult  - Difficult  - undecided  - Easy  - Very easy |
| 1. **Have you already participated in specialist training on the management of irAEs after ICI therapy?**   - Yes  - No |
| 1. **How often would you use the following sources of information for the management of side effects after ICI therapy?**   - App (very often, often, occasionally, rarely, never)  - Guideline (very often, often, occasionally, rarely, never)  - Internal action algorithm (SOP) (very often, often, occasionally, rarely, never)  - Adverse reaction register (very often, often, occasionally, rarely, never)  - (Online) further training (very often, often, occasionally, rarely, never)  - Training in your own institution (very often, often, occasionally, rarely, never) |
| 1. **Are you familiar with the phenomenon of “pseudoprogression” under ICI therapy?**   - Yes  - No |
| 1. **How many events of “pseudoprogression” have you experienced under ICI therapy in your clinical practice?**  - 0 - 1 - 2 - 3 - 4 - 5 - more than 5 |
| 1. **Do you use material for patient education treated with ICIs (multiple answers possible)?**   - Information sheets  - Brochures from the pharmaceutical manufacturers  - Information videos  - Reference to online material  - Other (please specify): “free text”  - None of the above |
| 1. **Would you like to receive more materials with information on side effect management under checkpoint inhibitor therapy for patient education?**   - Yes  - No |
| 1. **Which materials with information on irAE management under ICI therapy for patient education would you like to receive (multiple answers possible)?**   - None of the above  - Information sheets  - Brochures from the drug manufacturer  - Information videos  - Reference to online material  - Other (please specify) |
| 1. **When do you ask about symptoms of irAEs under ICI therapy?**   - At the start of therapy  - During the ongoing therapy  - After completion of therapy |
| 1. **Do you systematically ask for symptoms of irAEs of ICIs during your follow-up consultations?**   - Yes  - No |
| 1. **How long after the discontinuation of ICI therapy do you ask for possible symptoms of irAEs?**   - 0-1 month  - 1-3 months  - 3-6 months  - 6-12 months  - Longer than 12 months  - I never ask for these side effects |
| 1. **Please indicate your agreement or disagreement with each of the areas in which there is a need for improvement in the treatment of side effects of immune checkpoint inhibitors?**  - Interdisciplinary cooperation (Agree strongly, Agree, Agree somewhat, Undecided, Disagree somewhat, Disagree strongly Disagree) - Faster recognition of side effects (Agree strongly, Agree, Agree somewhat, Undecided, Disagree somewhat, Disagree strongly Disagree) - More training (Agree strongly, Agree, Agree somewhat, Undecided, Disagree somewhat, Disagree strongly Disagree) - Treatment Algorithms (Agree strongly, Agree, Agree somewhat, Undecided, Disagree somewhat, Disagree strongly Disagree) |
| 1. **What training needs do you have in the following areas?**   - Gastrointestinal side effects (Very much, Much, Moderately, Little, None)  - Dermatologic side effects (Very much, Much, Moderately, Little, None)  - Endocrine side effects (Very much, Much, Moderately, Little, None)  - Therapy management (Very much, Much, Moderately, Little, None)  - Application of the therapy (Very much, Much, Moderately, Little, None) |
