# Supplementary material for: The implementation and side effect management of immune checkpoint inhibitors in gynecologic oncology: a JAGO/NOGGO survey
Source: BMC Cancer. 2025 Jan 29;25:170. doi: 10.1186/s12885-025-13432-5 (PMC11776233; doi:10.1186/s12885-025-13432-5)
Supplement: Supplementary file 2 — Supplementary Material 2 [file 12885_2025_13432_MOESM2_ESM.docx]

**Supplementary table 1:** List of the states of origin of the respondents

| **State** | **n=** | **%** |
| --- | --- | --- |
| **Germany** | | |
| Baden-Wuerttemberg | 29 | 15,43% |
| Bavaria (Bayern) | 34 | 18,09% |
| Berlin | 32 | 17,02% |
| Brandenburg | 4 | 2,13% |
| Bremen | 0 | 0,00% |
| Hamburg | 4 | 2,13% |
| Hesse (Hessen) | 20 | 10,64% |
| Mecklenburg-Western Pomerania (Mecklenburg-Vorpommern) | 5 | 2,66% |
| Lower Saxony (Niedersachsen) | 11 | 5,85% |
| North Rhine-Westphalia (Nordrhein-Westfalen) | 19 | 10,11% |
| Rhineland-Palatinate (Rheinland-Pfalz) | 7 | 3,72% |
| Saarland | 5 | 2,66% |
| Saxony (Sachsen) | 4 | 2,13% |
| Saxony-Anhalt (Sachsen-Anhalt) | 0 | 0,00% |
| Schleswig-Holstein | 6 | 3,19% |
| Thuringia (Thüringen) | 0 | 0,00% |
| **Austria** | | |
| Graz | 1 | 3,57% |
| Lower Austria (Niederösterreich) | 2 | 7,14% |
| Upper Austria (Oberösterreich) | 1 | 3,57% |
| Salzburg | 1 | 3,57% |
| Styria | 6 | 21,43% |
| Tyrol | 4 | 14,29% |
| Vienna | 12 | 42,86% |
| **Switzerland** | | |
| Zug | 1 | 25,00% |
| Zurich (Zürich) | 3 | 75,00% |

**Supplementary table 2** How long after the completion of ICI therapy do you inquire about possible symptoms of irAEs?

|  | **%** | **n=** |
| --- | --- | --- |
| **0-1 months** | 5.2 | 11 |
| **2-3 months** | 11.0 | 23 |
| **4-6 months** | 16.2 | 34 |
| **7-12 months** | 21.0 | 44 |
| **longer than 12 months** | 37.6 | 79 |
| **I do not ask about side effects.** | 9.0 | 19 |

**Supplementary table 3** How frequently would you use the following informational resources for side effect management of ICI therapy?

|  | **very**  **often** | | **often** | | **occasionally** | | **rarely** | | **very**  **rarely** | | **total** | **weighted**  **average** |
| --- | --- | --- | --- | --- | --- | --- | --- | --- | --- | --- | --- | --- |
|  | n= | % | n= | % | n= | % | n= | % | n= | % | n= |  |
| **Mobile apps** | 63 | 29,6 | 21 | 31,5 | 77 | 17,4 | 71 | 8,0 | 45 | 13,6 | 213 | 3,6 |
| **Guidelines** | 57 | 26,3 | 20 | 44,2 | 51 | 21,7 | 73 | 5,5 | 68 | 2,3 | 217 | 3,9 |
| **Standard Operating**  **Procedure (SOPs)** | 67 | 31,2 | 17 | 40,0 | 42 | 13,5 | 68 | 5,1 | 86 | 10,2 | 215 | 3,8 |
| **Side effects registry** | 14 | 6,5 | 42 | 23,7 | 68 | 27,9 | 46 | 28,4 | 48 | 13,5 | 215 | 2,8 |
| **(Online) training** | 26 | 12,0 | 50 | 34,1 | 45 | 35,9 | 27 | 13,8 | 63 | 4,1 | 217 | 3,4 |
| **In-house training** | 42 | 19,6 | 2 | 36,4 | 14 | 27,1 | 63 | 7,5 | 135 | 9,3 | 214 | 3,5 |
